# Supplementary material for: Synthetic Polypeptides with Cationic Arginine Moieties Showing High Antimicrobial Activity in Similar Mineral Environments to Blood Plasma
Source: Polymers (Basel). 2022 May 2;14(9):1868. doi: 10.3390/polym14091868 (PMC9104764; doi:10.3390/polym14091868)
Supplement: Supplementary file 1 [file polymers-14-01868-s001.zip › polymers-1669605-supplementary.pdf]

Supporting Information

Synthetic Polypeptides with Cationic Arginine Moieties Showing High Antimicrobial Activity in Similar Mineral Environments to Blood Plasma

Kuen Hee Eom [1], Shuwei Li [1], Eun Gyeong Lee [1], Jae Ho Kim [2], Jung Rae Kim [1] and Il Kim [1,*]

[1] School of Chemical Engineering, Pusan National University, Busandaehag-ro 63-2, Geumjeong-gu, Busan 46241, Korea; reimreim@pusan.ac.kr (K.H.E.); lishuwei0325@pusan.ac.kr (S.L.); rud6063@pusan.ac.kr (E.G.L.); j.kim@pusan.ac.kr (J.R.K.)

[2] Department of Physiology, School of Medicine, Pusan National University, Busandaehak-ro, Mulgeum-eup, Yangsan-si 50612, Korea; jhkimst@pusan.ac.kr

* Correspondence: ilkim@pusan.ac.kr

Table of Contents

Figure S1 ^{1}H NMR spectra of *s*-p[(Cbz-Lys)$_{43}$]$_3$ (Entry 1 in Table 1) in DMSO-*d*$_6$.

Figure S2 ^{1}H NMR spectra of *s*-p[(Cbz-Lys)$_{31}$-*co*-(Phe)$_8$]$_3$ (Entry 2 in Table 1) in DMSO-*d*$_6$.

Figure S3 ^{1}H NMR spectra of *s*-p[(Cbz-Lys)$_{25}$-*co*-(Phe)$_{15}$]$_3$ (Entry 3 in Table 1) in DMSO-*d*$_6$.

Figure S4 ^{1}H NMR spectra of *s*-p[(Cbz-Lys)$_{31}$]$_3$ (Entry 4 in Table 1) in DMSO-*d*$_6$.

Figure S5 ^{1}H NMR spectra of *s*-p[(Cbz-Lys)$_{14}$-*co*-(Phe)$_7$]$_3$ (Entry 5 in Table 1) in TFA.

Figure S6 ^{1}H NMR spectra of *s*-p[(Cbz-Lys)$_{19}$-*co*-(Phe)$_{11}$]$_3$ (Entry 6 in Table 1) in DMSO-*d*$_6$.

Figure S7 ^{1}H NMR spectra of *s*-p[(Cbz-Lys)$_{16}$-*co*-(Phe)$_4$]$_3$ (Entry 8 in Table 1) in DMSO-*d*$_6$.

Figure S8 ^{1}H NMR spectra of *s*-p[(Cbz-Lys)$_{13}$-*co*-(Phe)$_9$]$_3$ (Entry 9 in Table 1) in DMSO-*d*$_6$.

Figure S9 ^{1}H NMR spectra of *mb*-p(Cbz-Lys)$_{24}$ (Entry 11 in Table 1) in TFA.

Figure S10 FTIR spectra of *s*-p(Lys)$_{21}$, *s*-p(Arg)$_{21}$, *l*-p[(Lys)$_{23}$-*co*-(Phe)$_7$], and *l*-p[(Arg)$_{23}$-*co*-(Phe)$_7$].

Figure S11 Determination of minimal inhibitory concentration (MIC) on *E. coli* cells with *l*-, *s*- and *mb*-polypeptides.

Figure S12 Determination of minimal inhibitory concentration (MIC) on *B. substilis* cells with *l*-, *s*- and *mb*-polypeptides.

Figure S13 Hemolytic activity of *mb*-p[(Arg)$_{23}$-*co*-(Phe)$_7$]$_8$ in fresh red blood cells of mice.

Supplementary figures

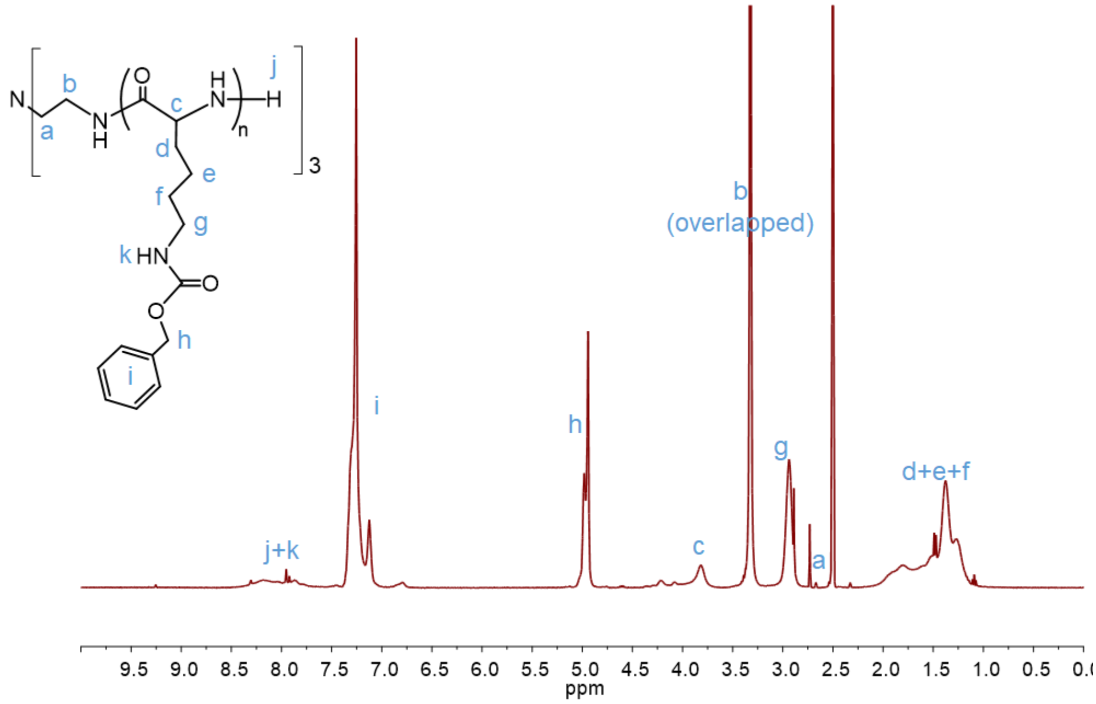

Figure S1 ^{1}H NMR spectra of *s*-p[(Cbz-Lys)$_{43}$]$_3$ (Entry 1 in Table 1) in DMSO-d_6.

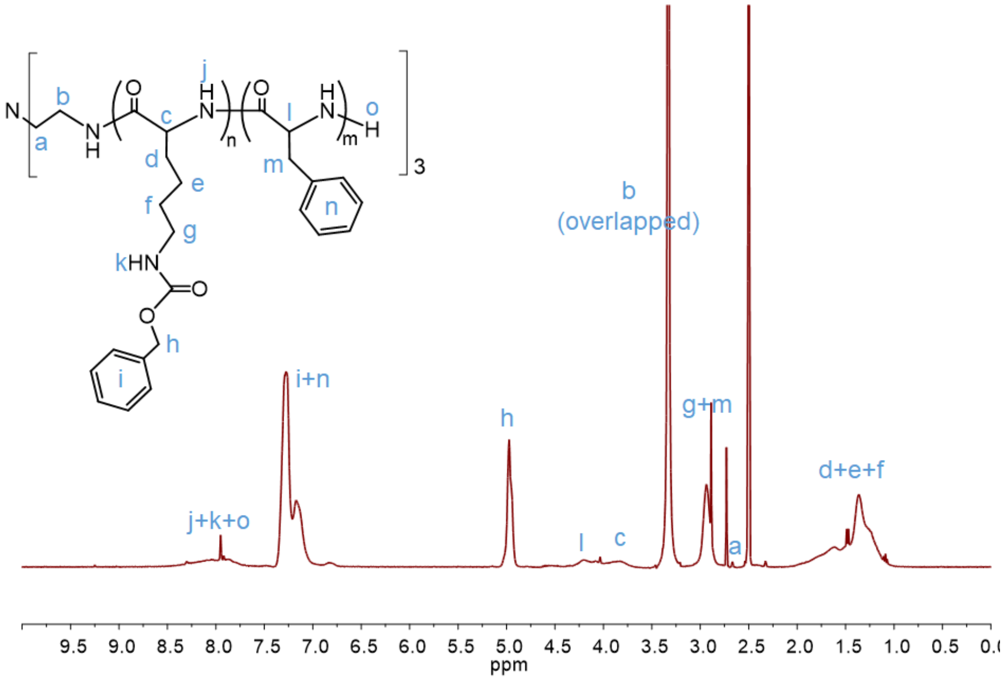

Figure S2 ^{1}H NMR spectra of *s*-p[(Cbz-Lys)$_{31}$-*co*-(Phe)$_8$]$_3$ (Entry 2 in Table 1) in DMSO-d_6.

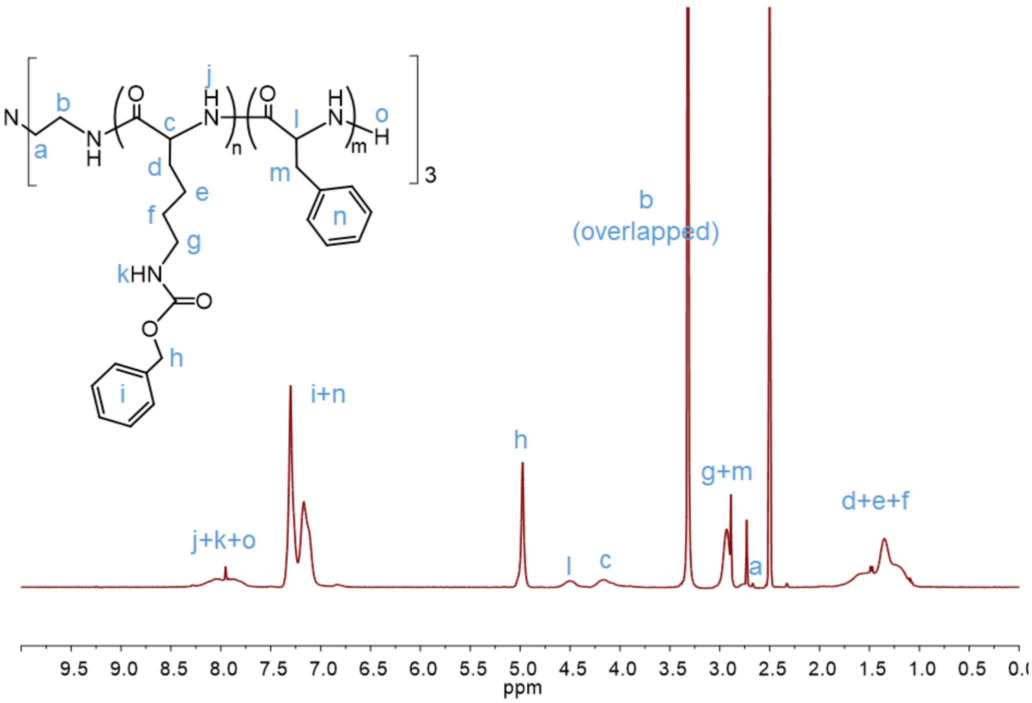

Figure S3 ^{1}H NMR spectra of *s*-p[(Cbz-Lys)$_{25}$-*co*-(Phe)$_{15}$]$_3$ (Entry 3 in Table 1) in DMSO-d_6.

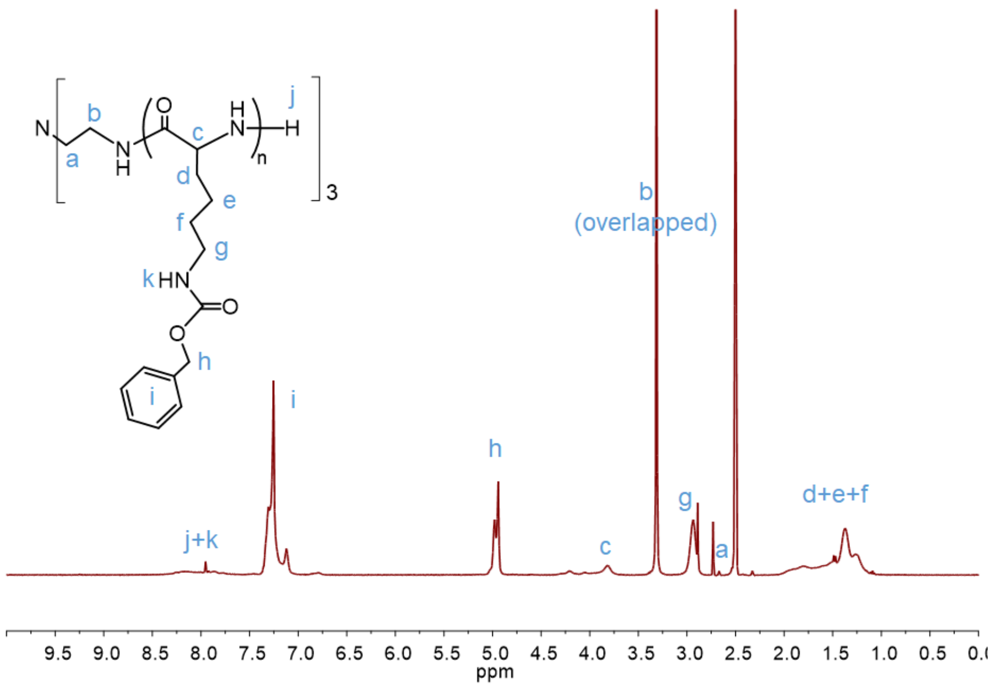

Figure S4 ^{1}H NMR spectra of *s*-p[(Cbz-Lys)$_{31}$]$_3$ (Entry 4 in Table 1) in DMSO-d_6.

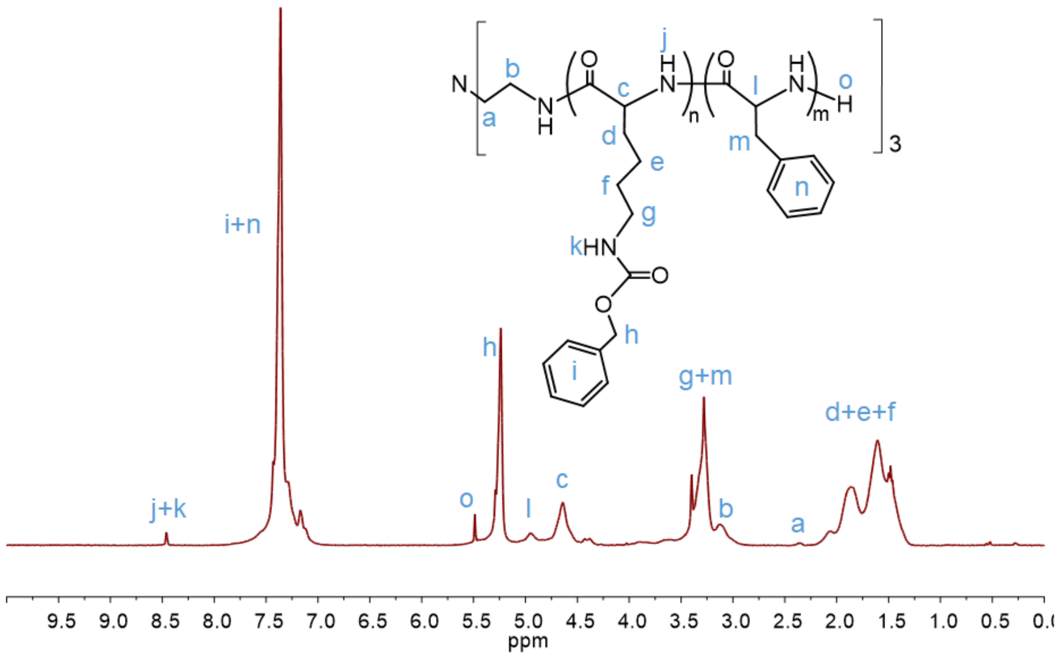

Figure S5 ^{1}H NMR spectra of *s*-p[(Cbz-Lys)$_{14}$-*co*-(Phe)$_{7}$]$_{3}$ (Entry 5 in Table 1) in TFA.

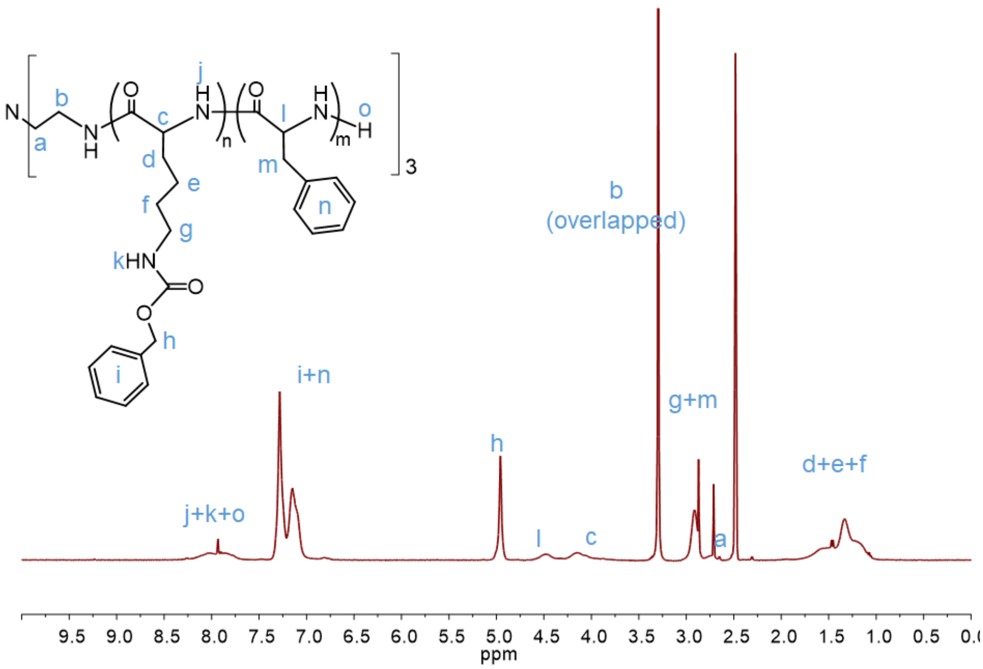

Figure S6 ^{1}H NMR spectra of *s*-p[(Cbz-Lys)$_{19}$-*co*-(Phe)$_{11}$]$_{3}$ (Entry 6 in Table 1) in DMSO-d_6.

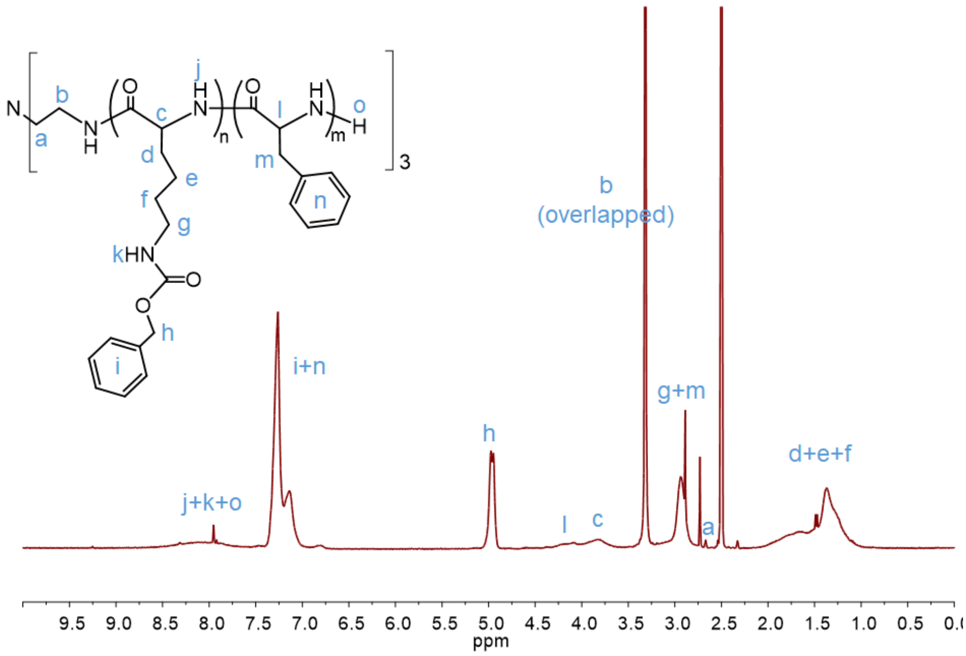

Figure S7 ^{1}H NMR spectra of *s*-p[(Cbz-Lys)$_{16}$-*co*-(Phe)$_{4}$]$_{3}$ (Entry 8 in Table 1) in DMSO-d_6.

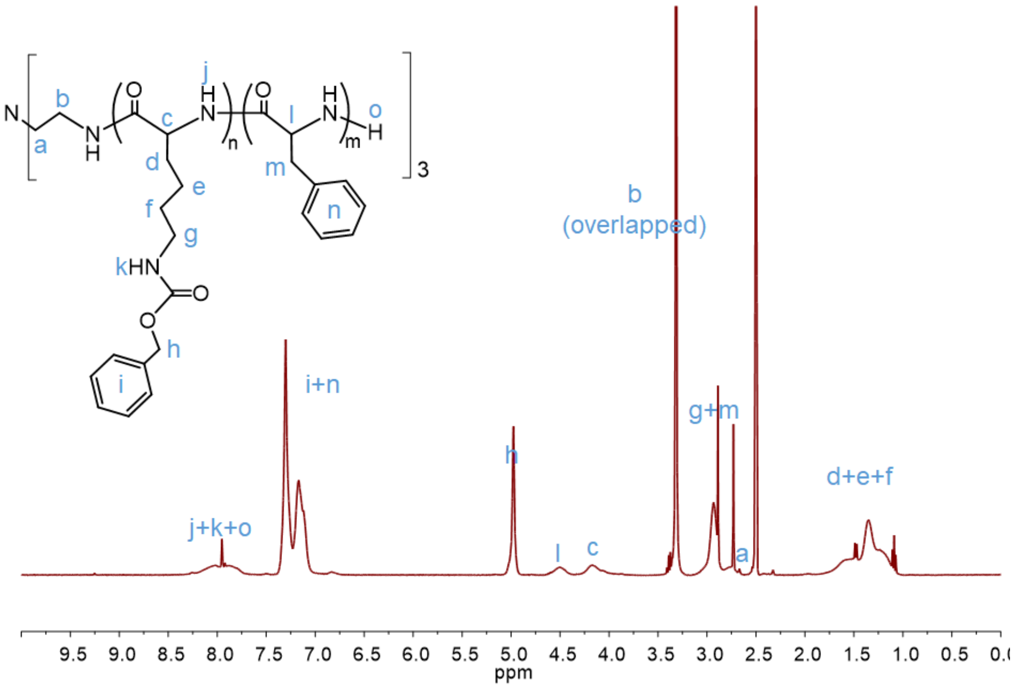

Figure S8 ^{1}H NMR spectra of *s*-p[(Cbz-Lys)$_{13}$-*co*-(Phe)$_{9}$]$_{3}$ (Entry 9 in Table 1) in DMSO-d_6.

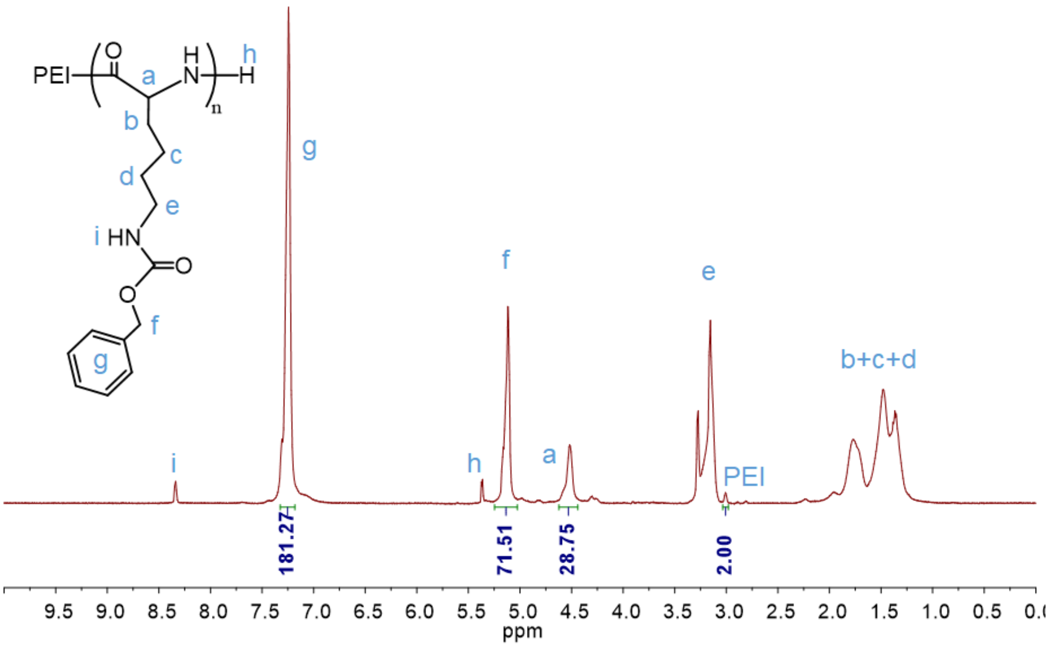

1

Figure S9 ^{1}H NMR spectra of *mb*-p(Cbz-Lys)$_{24}$ (Entry 11 in Table 1) in TFA.

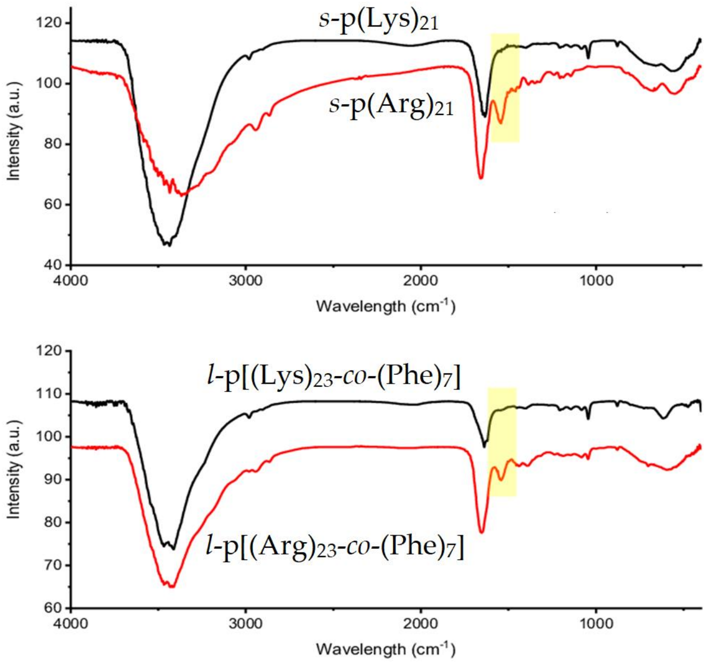

Figure S10. FTIR spectra of *s*-p(Lys)$_{21}$, *s*-p(Arg)$_{21}$, *l*-p[(Lys)$_{23}$-*co*-(Phe)$_7$], and *l*-p[(Arg)$_{23}$-*co*-(Phe)$_7$].

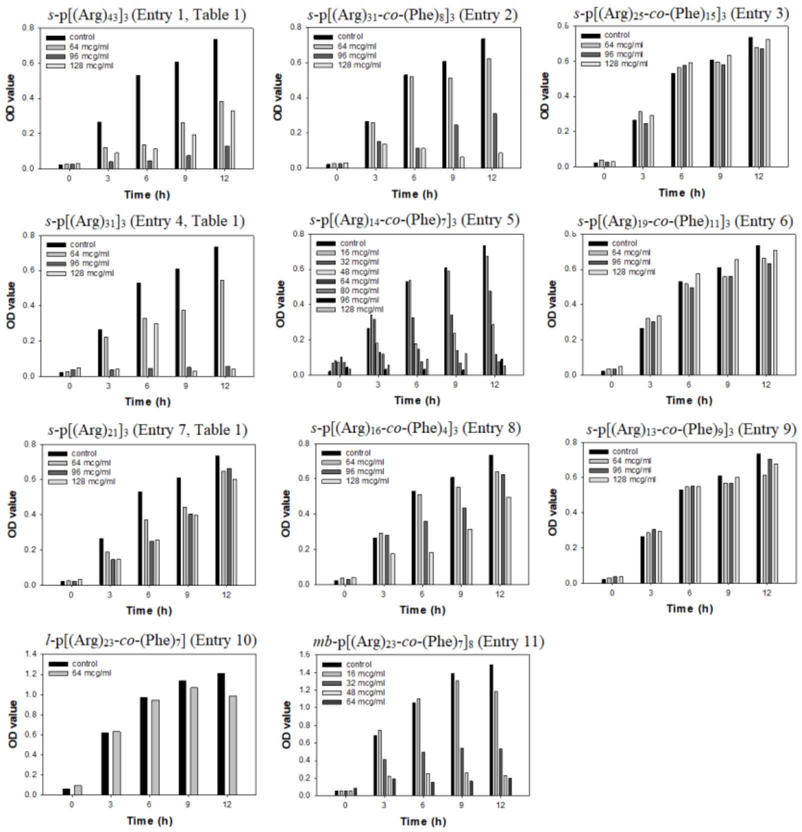

Figure S11. Determination of minimal inhibitory concentration (MIC) on *E. coli* cells with *l*-, *s*- and *mb*-polypeptides.

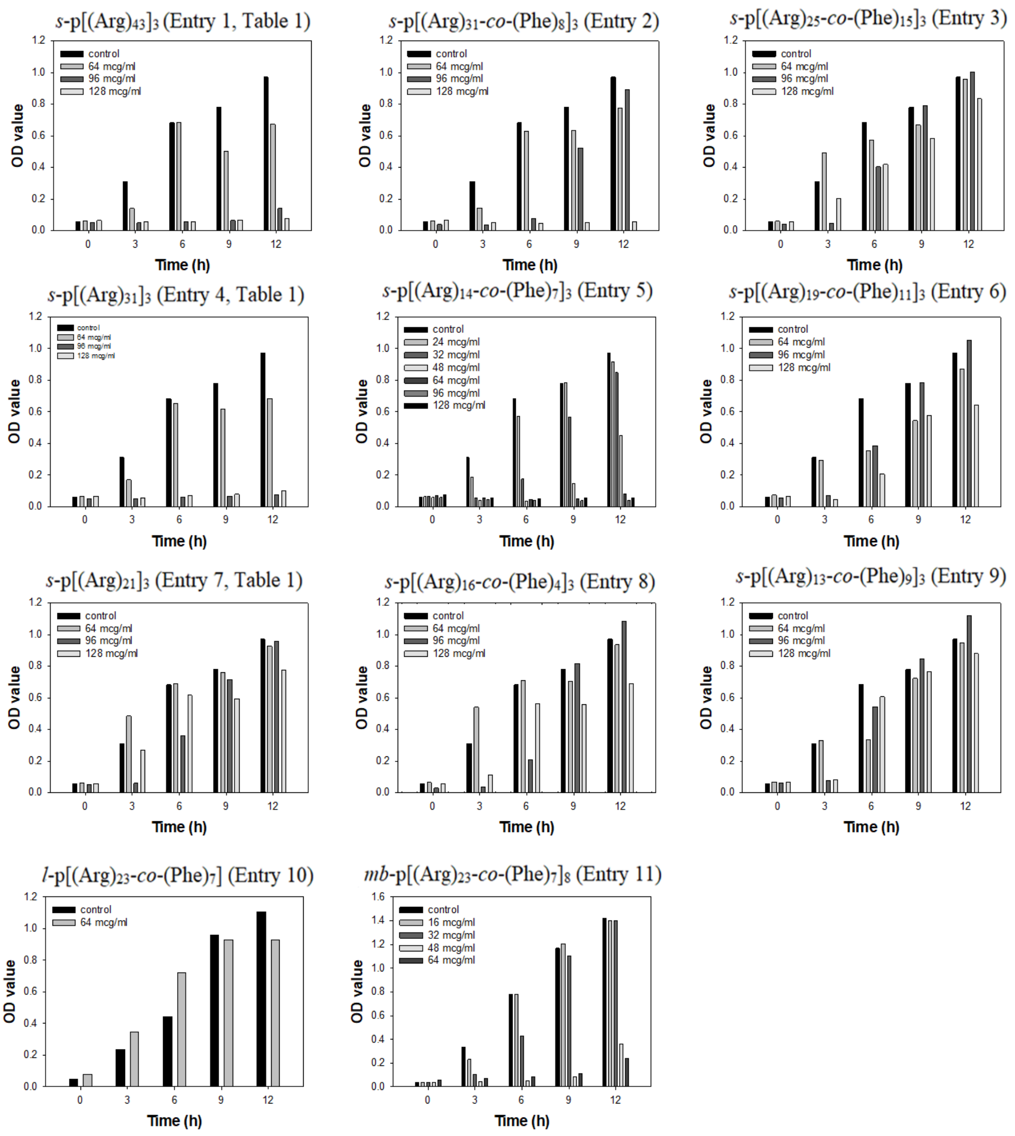

Figure S12. Determination of minimal inhibitory concentration (MIC) on *B. substilis* cells with *l*-, *s*- and *mb*-polypeptides.

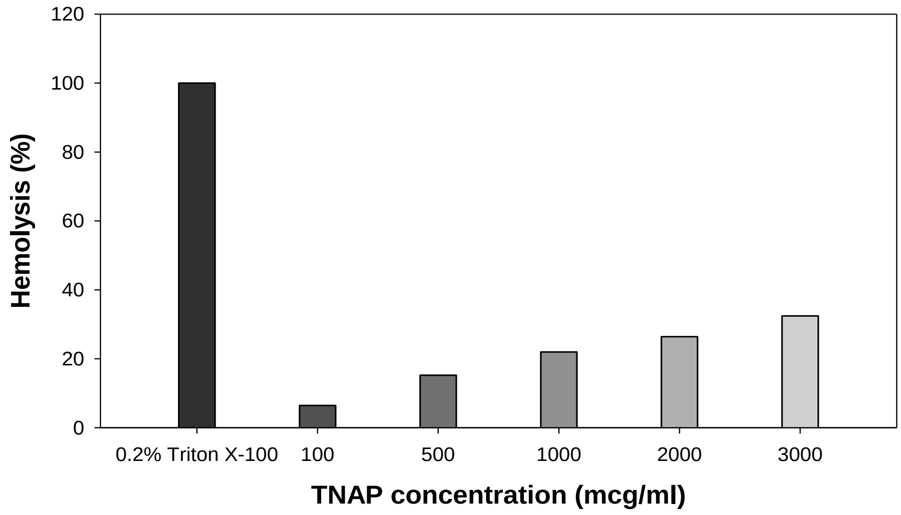

Figure S13. Hemolytic activity of *mb*-p[(Arg)$_{23}$-*co*-(Phe)$_7$]$_8$ in fresh red blood cells of mice.
